# Views of healthcare professionals on complementary and alternative medicine use by patients with diabetes: a qualitative study

**DOI:** 10.1186/s12906-024-04385-6

**Published:** 2024-02-09

**Authors:** Abdulaziz S. Alzahrani, Sheila M. Greenfield, Sunil Shrestha, Vibhu Paudyal

**Affiliations:** 1 School of Pharmacy, College of Medical and Dental Sciences, Sir Robert Aitken Institute for Medical Research, University of Birmingham, Birmingham, B15 2TT UK; 2 Institute of Applied Health Research, University of Birmingham, Birmingham, UK; 3 School of Pharmacy, Monash University Malaysia, Subang Jaya, Selangor 47500 Malaysia

**Keywords:** Complementary and alternative medicine, Diabetes, Healthcare professionals

## Abstract

**Background:**

Recent estimates indicate that a significant proportion of diabetic patients globally, up to 51%, are utilizing complementary and alternative medicine (CAM). To improve patient-provider communication and optimize prescribed treatments, healthcare professionals (HCPs) must understand the factors associated with CAM use among diabetic patients. There is a dearth of literature on HCPs perspectives on CAM use by diabetic patients. This study explored HCPs knowledge, perspective, and views on their diabetic patients’ use of CAM.

**Methods:**

Qualitative study using one-to-one semi-structured interviews conducted with 22 HCPs involved in the care of diabetic patients (6 endocrinologists, 4 general practitioners, 4 nurses and 8 pharmacists). Participants were recruited through general practices, community pharmacies and a diabetic centre in Saudi Arabia. Data were analyzed using thematic analysis.

**Results:**

Five key themes resulted from the analysis. HCPs generally demonstrated negative perceptions toward CAM, particularly regarding their evidence-based effectiveness and safety. Participants described having limited interactions with diabetic patients regarding CAM use due to HCPs’ lack of knowledge about CAM, limited consultation time and strict consultation protocols. Participants perceived convenience as the reason why patients use CAM. They believed many users lacked patience with prescribed medications to deliver favourable clinical outcomes and resorted to CAM use.

**Conclusions:**

HCPs have noted inadequate engagement with diabetic patients regarding CAM due to a lack of knowledge and resources. To ensure the safe use of CAM in diabetes and optimize prescribed treatment outcomes, one must address the communication gap by implementing a flexible consultation protocol and duration. Additionally, culturally sensitive, and evidence-based information should be available to HCPs and diabetic patients.

**Supplementary Information:**

The online version contains supplementary material available at 10.1186/s12906-024-04385-6.

## Background

The World Health Organization (WHO) defines complementary and alternative medicine (CAM) as a “*broad set of health care practices that are not part of that country’s own tradition or conventional medicine and are not fully integrated into the dominant health-care system*” [1]. The use of CAM for diabetes self-management is prevalent globally, estimated at 51% [2]. The prevalence of CAM use varies between countries, ranging between 8 and 89%. Use by diabetic patients’ in Saudi Arabia is among the highest worldwide, ranging between 25.8 and 64% [2]. It is estimated that up to two-thirds of diabetic patients who use CAM do not discuss their use with their healthcare professionals (HCPs) [2].

In the context of this study, CAM encompasses a broad range of healthcare practices and therapies that are not considered part of conventional Western medicine. In the Saudi Arabian healthcare setting, CAM includes but is not limited to herbal remedies, traditional healing practices, acupuncture, spiritual healing, and dietary supplements [2–4]. CAM encompasses a spectrum of non-mainstream approaches that aim to enhance well-being, manage symptoms, or address underlying health concerns outside the scope of conventional medicine. Several texts in Islamic literature recommend approaches to treating disease, especially honey, cupping, and ruqya [5], but do not offer specific advice for treating diabetes. The detailed of CAM practices in the Saudi Arabian Context is available in Table 1.

**Table 1:** CAM practices in the Saudi Arabian context

Recommended Approach	Definition/Comments
Ruqya	Ruqya is a practice that is well-known in Islamic culture [6]. Ruqya is a form of exorcism usually performed by a sheikh who recites verses of the Qur’an and prayers for the patient.
Cupping	Cupping therapy involves using a small vessel that creates a vacuum force when applied to the surface of skin. This technique targets a particular area, enhancing metabolic activity, boosting immune function, and stabilizing blood biochemistry by promoting blood circulation and autologous healing substances [7, 8].
Honey	Honey is highly regarded in Islamic culture for treating all sorts of diseases [9].
Cauterization	Cauterization or cutaneous cautery refers to using a hot iron rod and applying it to parts of the body, causing tissue burns and damage in an attempt to treat diseases [10].

HCPs are expected to support patient self-care and self-management to improve disease outcomes and quality of life [11]. To maximize disease outcomes for patients with diabetes, the HCPs responsible for developing and monitoring diabetes management plans must possess comprehensive knowledge of their patients’ self-management practices, including CAM use. For example, patients might ultimately give up on conventional medicine and use CAM as an alternative to their prescribed medicines, increasing the risk of developing diabetes-related complications [12]. Moreover, patients using CAM with their prescribed medicines could encounter CAM-drug interactions, particularly with herbal products, that could interfere with the effectiveness of prescribed medicines and lead to harmful outcomes [13]. Thus, HCPs must have an open dialogue with their patients regarding the benefits and risks of CAM use as a part of their diabetes management plan.

Existing literature exploring HCPs’ perspectives on the use of CAM by diabetic patients is limited. A study conducted in Uganda investigated diabetic patients’ CAM use from the perspective of HCPs [14]. Another study conducted in Saudi Arabia with type 2 diabetes patients and physicians only focused on herbal medicines [15]. Both studies used cross-sectional, closed-ended questionnaires and showed different results. The first reported that HCPs had a relatively positive attitude towards their patients’ CAM use, while the second described that most participating HCPs had a negative attitude towards their diabetic patients’ herbal medicine use. This study used a qualitative approach to gain a deeper understanding of HCPs’ perspectives on their diabetic patients’ CAM use. Such research is important because it can inform patient management and treatment planning and improve patient outcomes. Qualitative study design can provide a deeper understanding of HCPs’ attitudes towards CAM and the reasons behind their beliefs, contributing to the growing body of research on integrating CAM therapies into mainstream healthcare.

This study aimed to explore the views and experiences of HCPs regarding the use of CAM for diabetes self-management by diabetic patients in Saudi Arabia.

## Method

The study used one-to-one qualitative, in-depth, semi-structured interviews with HCPs working with diabetic patients in Al-Baha, Saudi Arabia [16]. Al-Baha is situated in the south-west of Saudi Arabia, a popular summer destination for Saudis because of its cool climate. Consequently HCPs, in this region come into contact with a diverse segment of the Saudi population through their interactions with visitors. The data for this study were collected from three cities within the Al-Baha region: Al-Baha City, Almandaq, and Baljurashi.

### Study setting

Due to the restrictions during the COVID-19 pandemic, interviews with participants were conducted by telephone [17]. The participants selected their interview date and time and were asked to choose an appropriate and private location to receive the interview call to protect their privacy and confidentiality. The researcher also called from a designated private room to protect participants’ privacy. There were no participants who declined to take part in the study. Piloting was conducted with some PhD students at the University of Birmingham pharmacy school.

### Sampling and recruitment

The study included HCPs directly responsible for providing medical care to diabetic patients. Potential participants were identified through the Saudi Ministry of Health directory and contacted by phone by calling healthcare facilities in the Al-Baha region of Saudi Arabia. Participants were recruited from hospitals, general practices, community pharmacies and a diabetic centre. Invitation letters were sent to potential participants via email that candidates provided to the researcher. Participants were asked to confirm their eligibility to participate in the study by providing evidence of their professional registration and describing their specific role in the care of diabetic patients. A purposive maximum variation sample was used [18] to ensure the sample was as diverse as possible, aiming to include HCPs with different roles in patient care. Ten participants were initially recruited, and then a further 3 participants were interviewed until data saturation was achieved at 22 interviews, i.e., no new themes emerged [19, 20].

### Data collection

All interviews were carried out by AA, a PhD student in pharmacy at the University of Birmingham, UK and a native of the Al-Baha region in Saudi Arabia. Interviews lasted an average of 45 minutes. The interview schedule was developed based on previous findings in the literature and the authors’ previous work [2–4]. The interview schedule is available in Supplementary File 1. Participants were given a choice for the interview to be carried out in Arabic or English. Based on the participants’ preferences, 4 interviews were conducted in English and 18 in Arabic. The author, a native Arabic speaker, conducted and directly oversaw interviews, ensuring nuanced understanding. Interviews were recorded and transcribed in the original language of the interview. In ensuring methodological rigor, our qualitative study incorporated member checking, peer debriefing, and expert consultation for credibility; maintained an audit trail and involved multiple researchers for confirmability; practiced reflexivity for authenticity; and documented study procedures transparently for dependability. The interview transcripts of the interviews conducted and transcribed in Arabic were translated into English before data analysis using a UK-based University of Birmingham-approved professional translation service. Proficient in Arabic and English, the author played a crucial role in verifying the authenticity of translations. Rigorous verification thoroughly compared translated text with original interviews, confirming accurate conveyance. In cases of ambiguity, direct consultation with participants in an iterative feedback loop ensured the final translation reflected participants’ perspectives.

### Data analysis

Data were analyzed using inductive thematic analysis [21]. A sample of interview transcripts were independently reviewed and coded by two authors, AA, and VP, to ensure consistency and rigor in the analysis. Parts and segments of transcripts were coded with similar codes and grouped to form categories based on their similarities. The categories were then grouped into themes. The themes developed were then plotted in thematic illustrations summarising all the resulting themes. Discrepancies in coding were discussed and resolved through consensus or consultation with a third author, SG, where needed.

## Results

### Sample characteristics

Twenty-two HCPs who took part consisted of 8 pharmacists, 6 endocrinologists, 4 general practitioners and 4 nurses. Participants were recruited from a diabetic centre, hospitals, general practices and pharmacies (Table 2). Participants had experience caring for diabetes patients ranging between 2 and 30 years. At the beginning of each interview, information about the participants’ years of experience and their involvement in the care of diabetic patients was collected.

**Table 2:** Participants information

Participant	Profession	Years of experience	Place of work
Participant 1	Pharmacist	2	Diabetes Centre
Participant 2	Pharmacist	13	Diabetes Centre
Participant 3	Pharmacist	6	Community Pharmacy
Participant 4	Pharmacist	14	Community Pharmacy
Participant 5	Pharmacist	12	General Practice
Participant 6	Pharmacist	11	General Practice
Participant 7	Pharmacist	7	Hospital Pharmacy
Participant 8	Pharmacist	4	Hospital Pharmacy
Participant 9	Endocrinologist	25	Diabetes Centre
Participant 10	Endocrinologist	15	Diabetes Centre
Participant 11	Endocrinologist	30	Diabetes Centre
Participant 12	Endocrinologist	28	Diabetes Centre
Participant 13	Endocrinologist	9	Diabetes Centre
Participant 14	Endocrinologist	6	Diabetes Centre
Participant 15	General Practitioner	12	General Practice
Participant 16	General Practitioner	10	General Practice
Participant 17	General Practitioner	15	General Practice
Participant 18	General Practitioner	6	General Practice
Participant 19	Nurse	9	Diabetes Centre
Participant 20	Nurse	11	General Practice
Participant 21	Nurse	2	General Practice
Participant 22	Nurse	13	General Practice

### Key themes and summary of the findings

Five main themes were identified, relating to HCPs’ attitudes toward their diabetic patients’ CAM use, perspectives on the reasons for diabetic patients’ use of CAM, insight into their views on the safety and effectiveness of CAM for managing diabetes, cultural perspectives around certain CAM types and discussing CAM use with the diabetic patients (Table 3).

**Table 3:** Key themes and subthemes

No.	Themes	Sub-themes
1	Healthcare professionals’ attitude toward their diabetic patients’ CAM use	General attitude towards using CAM for the management of diabetes
		Healthcare professional attitude towards patients’ CAM use
2	Healthcare professionals’ perspectives on the reasons for diabetic patients’ use of CAM	Lay advice and referrals
		Patients’ feelings and expectations
		Countering side effects of conventional medicines
		Convenience
3	Healthcare professionals’ views on the safety and effectiveness of CAM for managing diabetes	CAM safety and effectiveness
		Determining CAM effectiveness
		Reporting CAM-related side effects and adverse events
4	Cultural perspectives around certain CAM types	Ruqya
		Cupping
		Honey
		Cauterisation
		Folk healers
5	Discussing CAM use with the diabetic patients	Frequency and nature of discussions
		Barriers to effective communications around CAM

### Healthcare professionals’ attitude toward their diabetic patients’ CAM use

#### General attitude towards using CAM for the management of diabetes

Participating HCPs, in general, had a negative attitude towards using CAM to manage diabetes. They perceived CAM as ineffective in managing diabetes and possibly harmful. One participant stated that even if a specific type of CAM is promising, CAM lacks adequate research and development procedures to establish its effectiveness. Participants also described variations in components of herbal treatments depending on environmental factors and extraction procedures.

“A plant can have the same shape, the same name and belong to the same family. However, it might produce two different components if it was planted in different places. If it were planted in one part of the mountain, it could contain a different component if it was planted in another part of the mountain … but if we extract the chemical compound from the herb, and it was possible to measure the concentration of the drug and the concentration of the substance, such as digoxin, then it is acceptable because it went through the same stages that chemical drugs go through. However, the way alternative medicine and traditional medicine are currently, I do not agree with them” [Participant 2, Pharmacist, 13 years’ experience].

### Healthcare professional attitude towards patients’ CAM use

Some participants were more lenient about the use of CAM for the management of diabetes. They would not mind their patients using some known types of herbs such as cinnamon and fenugreek. They perceived these as well-known and harmless. HCPs also stated that CAM should be taken in small quantities as using large amounts of CAM frequently is unacceptable. Participants mentioned that their patients often brought herbal products with unknown ingredients from countries such as China, India, Indonesia, Egypt, and Ukraine. HCPs did not usually approve of the types of herbs their patients brought from abroad.

“Some patients brought strange herbs from China and India; the ingredients were unknown, and I did not advise them to use them because this could have unknown complications. We advise them to use alternatives from well-known companies whose content is known” [Participant 10, Endocrinologist, 15 years’ experience].

Most participants described never having advised or prescribed CAM to their patients in clinical settings. However, two participants said they might consider recommending CAM when patients desperately sought a CAM product.

“I use honey on patients. I have honey that I brought from a trusted source. Of course, we are prohibited from using honey in the clinic, but I use it as a desperate measure for hopeless cases” [Participant 20, Nurse, 11 years’ experience].

### Healthcare professionals’ perspectives on the reasons for diabetic patients’ use of CAM

#### Lay advice and referrals

Participants reported that their diabetic patients often referred to receiving much advice from others, mainly family, friends, and other diabetic patients. HCPs were aware of CAM-related advertisement materials circulated to patients through WhatsApp. HCPs believed these materials strongly influenced patients’ decision to try CAM. Participants also reported that direct-to-patient advertisements conducted by dietary supplement sales representatives influenced diabetic patients’ decision to use CAM.

“Some companies’ representatives do come here promoting some products to patients claiming they are good for diabetes such as preparations containing vitamins and iron. But to avoid side effects I always tell them not to promote it to any type of patient” [Participant 9, Endocrinologist, 25 years’ experience].

### Patients’ feelings and expectations

Patients’ feelings of boredom and tiredness from diabetes management routines were deemed by participants to have forced them to seek alternative treatments to find something that would provide a faster recovery. Moreover, because of the reputation of diabetes having ‘horrible’ outcomes such as neuropathy and nephropathy, patients were often deemed desperate and therefore open to trying anything. One participant stated that patients usually could not control their blood glucose level after initial diagnosis as the first few weeks were usually for dose adjustment. However, they described that patients often jumped to the conclusion that conventional medicines did not work for them and used CAM as an alternative diabetes management approach. Participants expressed that patients were aware that conventional medicines do not offer a permanent cure for diabetes; therefore, they tried CAM to find a cure. Some patients, especially ones with uncontrolled diabetes, used CAM as they believed it had a synergistic effect that potentiates the effect of conventional medicines, or they used CAM as the last resort after everything else failed, according to participants. A participating pharmacist said that patients used some plants with a highly bitter taste, thinking they could lower blood glucose because of their bitterness. They attributed that to the patients’ understanding of the disease based on its name. Diabetes is translated as “sugar disease” in Arabic. The name sugar disease has caused a widespread belief that sweets cause diabetes, so it is believed that opposite, bitter things, would reverse the disease.

“In some cases, the doctor will prescribe a medication based on one’s condition, and they use it but see no benefit in the first week. They are supposed to follow the treatment given to them by the doctor intensively for the first month to be able to adjust the dose. However, they jump into the conclusion that the doctor could not help them and does not know anything. Then they resort to other solutions” [Participant 2, Pharmacist, 13 years’ experience].

### Countering side effects of conventional medicines

Participants mentioned that some of their patients used CAM to counter the side effects of conventional medicines. Folk healers had convinced patients that CAM they provided would not have the side effects that traditional medicines cause. A nurse participant said some patients were convinced prescribed treatments cause sexual health issues and used CAM instead to avoid this, and even though this was a major concern for patients, they were reluctant to discuss it.

“Patients use CAM to avoid the effect of diabetes tablets on them sexually, which is the most important reason…I do not know; I just hear hints from patients. It is impossible for someone to be open about it. But I expect that they read and hear about it, and then they become psychologically affected by it … They use it as an alternative for treating diabetes because they believe that conventional medications cause erectile dysfunction.” [Participant 20, Nurse, 11 years’ experience].

### Convenience

Many participants mentioned that diabetic patients resorted to CAM to avoid following a strict diet and regular exercise. They said some diabetic patients would like to continue their sedentary lifestyle and eat what they want without worrying about the effect on blood glucose levels.

Convenience of location was a perceived factor for using CAM by patients in rural areas, as hospitals were far away, and appointments were difficult to obtain. Therefore, patients resorted to CAM and folk healers to manage their diabetes.

“Patients complain that hospitals are overcrowded, appointments are far apart, and it is difficult to communicate with a doctor. They find it easier to go and see someone else, pay them 100 riyals only and end of story…The further the person lives from the city, the more they are inclined to use alternative medicine. It is easier for people living in rural areas to go and see folk healers than to go to the city, which can be 60km away.” [Participant 4, Pharmacist, 14 years’ experience].

### Healthcare professionals’ views on the safety and effectiveness of CAM for managing diabetes

#### CAM safety and effectiveness

Participants’ views on the safety of CAM were subjective according to whether the type of CAM used was well-known. They considered CAM types available in the market, such as cinnamon and fenugreek, to be safe. Some participants perceived them as natural and, therefore safe. However, they were reluctant to judge the safety of less popular CAM types such as other herbal medicines, homoeopathy, or acupuncture.

Participants had a neutral attitude toward the effectiveness of CAM. They did not dismiss the potential of CAM being effective for diabetes, but at the same time, they did not believe that it is effective, as currently there was no evidence of this. According to the HCPs, they had limited experience with the efficacy of CAM for diabetes. On rare occasions, they described having observed slight improvements in their patients. However, one pharmacist expressed scepticism about the reported effectiveness of CAM for diabetes, even when patients claimed to have lower blood glucose levels. The pharmacist cautioned that “correlation does not imply causation” and questioned all indications of CAM effectiveness reported by patients.

“I believe that they are some kind of medicines like which are completely based extracted from the plant or the natural organic products only they must have some beneficial effects but there are many fake medicines as well so patients should be careful about what they are purchasing, and they should know the ingredients from whoever is giving them. I’m telling them again and again to just ask the person whoever is giving and making them the main of the ingredients at least you should know the ingredients because you don’t know they just give you and you don’t know what you’re using.” [Participant 18, General Practitioner, 6 years’ experience].

### Determining CAM effectiveness

Some participants stated that there was no feasible and reliable way to measure the effectiveness of CAM. They said the only remotely credible way was to observe improvement reported by patients in their glycaemic control. They described that many other confounding factors, such as psychological effects and changes in diet or physical activities, could play a role in that improvement.

“I cannot be sure. The improvement may be that she adhered to the treatment better or that the diet was better. If it happened during a period when I changed her treatment and noticed an improvement, then I can judge that the improvement was caused by the new treatment. For this patient we would have made a dose adjustment for her from the previous time. Therefore, it cannot be judged that the mix was the reason for the improvement.” [Participant 13, Endocrinologist, 9 years’ experience].

### Reporting CAM-related side effects and adverse events

None of the participants in this study reported any side effects of CAM to the Saudi National Pharmacovigilance and Drug Safety Centre. One reason was that they had never witnessed side effects or adverse events associated with CAM that were worth reporting. Furthermore, patients had never raised concerns about side effects and adverse events attributed to CAM, so professionals would remain unaware of any issues that should be reported.Moreover, participants did not think that side effects or adverse events associated with CAM use for diabetes should be reported, as they thought only side effects associated with medicines should.

“If it happens, it is mostly an allergy to the ingredients, but it is rare. For example, you ask a patient about the cause of allergy, and they say that he used such and such. For example, aloe vera, some people are allergic to it … it is not a well-known product on the market for me to report it. For example, some people are allergic to eggs, it makes no sense that I report that this person is allergic to eggs. Aloe vera is found anywhere, and it is not a medicinal product. If there was a well-known promoted product and I noticed side effects, then I report it.” [Participant 7, Pharmacist, 7 years’ experience].

### Cultural perspectives around certain CAM types

Participants suggested that the recommendations in Islamic literature influenced diabetic patients’ choice of CAM use and described that many patients interpret the recommendations in Islamic literature as relevant to diabetes management. They stated that diabetic patients also use CAM types not recommended in Islamic literature but popular in local Saudi cultures, such as cauterization and folk healers.

#### Ruqya

Participants were aware of patients who believed that the evil eye hit them and that diabetes they developed was not a disease on its own but rather a symptom of the evil eye, and once the evil eye is cured through ruqya, diabetes will be cured as well.

Some participants expressed concerns that they knew that sheikhs often advised patients to stop using diabetes medications and rely on ruqya alone, which causes low adherence and results in uncontrolled diabetes. An endocrinologist who participated in the study expressed apprehension regarding patients’ beliefs about the cause of diabetes. The endocrinologist noted that if patients believe that the evil eye is the root cause of their diabetes, they may discontinue their prescribed medications, assuming that conventional medicine will be ineffective. This could result in uncontrolled diabetes, leading to various related problems.

Another participating endocrinologist explained that this belief was more prominent among parents with young diabetic children than adult diabetic patients. She explained that the reason for that was that when children are first diagnosed with diabetes, they start with the treatment using insulin injections, unlike adults who start on oral medications. The parents saw this as invasive and found it difficult to accept or comprehend the situation. Therefore, parents resorted to explaining that their children’s diagnosis of diabetes is associated with the evil eye. However, some HCPs believed that ruqya might benefit other diseases, not diabetes. A participating pharmacist said that if the evil eye causes diabetes, ruqya might slow down diabetes progression and help prevent complications but never cure it.

“Some sheikhs convince them that they suffer from evil eye or some type of black magic in the tummy then they stop taking the medication and resort to spiritual treatment and oils etc. Then they believe they are getting better, but it is an illusion, when in fact their condition is getting worse” [Participant 6, Pharmacist, 11 years’ experience].

### Cupping

A participating pharmacist doubted that cupping, in the way it is currently performed would, impact diabetes. The participant explained that there were no specialized, fully qualified people who could properly perform cupping. Another participating pharmacist said that cupping might benefit external illnesses such as headaches and muscle aches but would not affect internal diseases such as diabetes. A participating nurse said that cupping had a temporary effect on diabetes; once that initial effect wears off, the condition returns to how it was; hence, it was concluded that cupping is not a viable treatment option. Participants expressed the difficulties of advising against CAM types with a religious background.

“Cupping has religious roots. It is difficult to convince patients that cupping has no effect.” [Participant 4, Pharmacist, 14 years of experience].

“I try to respect the socioeconomic and traditional practices of my patients.” [Participant 9, Endocrinologist, 25 years’ experience].

#### Honey

Participants described honey as one of the CAM types favoured by diabetic patients, i.e., patients who like to use it. Participants also said that honey was sometimes effective, especially for diabetic foot. Participants expressed that only pure and natural honey could be beneficial and even allowed honey to be used for clinic patients.

“I noticed that honey is somewhat more useful than the ointments which are distributed to them from the Centre or hospital in speeding up wound healing if the honey is pure.” [Participant 21, Nurse, 2 years’ experience].

“Honey is really good it’s like kind of expensive I don’t know how much they get it for I don’t know how much kilo for 300 riyals, something like, but it is very it is very beneficial so if they’re bringing the honey with them, we have told the nurses OK fine if they want and if they are satisfied with it OK” [Participant 17, General Practitioner, 15 years’ experience].

#### Cauterization

Participants described that some patients went to people who perform cauterization in the hope of curing diabetes. The patients got cauterized on their hands, feet, chin, knees and other parts of their bodies. One general practitioner said that although patients did not get any results from it, they seemed satisfied and happy about being cauterized. A pharmacist explained that cauterization and acupuncture were similar to a certain extent. The participant assumed both CAM types to revive the pancreas, but he said that was impossible as the diabetic patient’s pancreatic cells were virtually destroyed beyond repair. Participants expressed concern about the slow healing of wounds in diabetic patients who underwent cauterization and the possibility of getting the cauterization wounds infected.

“Sometimes they use cauterisation on a hand or a leg to treat diabetes, but it causes other problems and end up in an endless maze which they are better off without … Diabetic patients see people for cauterisation, and then gets into the trouble of cauterising and changing dressings daily while there was no need for the hassle until the cauterisation wound was heals.” [Participant 22, Nurse, 13 years’ experience].

#### Folk healers

Participants shared their views and experiences about their diabetic patients visiting folk healers. They expressed their concerns that folk healers were not trained or specialized. They were also concerned that there was no way to tell which folk healers knew what they were doing, and which were frauds, only claiming to be able to treat diabetes for financial exploitation. They also explained that patients in rural areas saw more folk healers than in cities. Nonetheless, they reported that patients sometimes travel to seek the advice of folk healers. Healthcare providers explained that some folk healers claimed they could permanently cure diabetes, while others targeted people with a family history of diabetes who had not been diagnosed with diabetes yet, claiming they could provide treatments that would prevent its development. Participants expressed their lack of ability to advise patients about what the folk healers had provided them with, as the folk healers did not reveal their “secrets of the trade”, and patients were provided with remedies with unknown content.

“Folk healers give them mixes which I do not know. They do not give them to the patients to apply them. Instead, the folk healers apply them themselves by giving the patients appointments. I do not know their ingredients. They tell the patients that they cannot give them the herbs to use at home because it is a secret recipe.” [Participant 20, Nurse, 11 years’ experience].

“Diabetes in general, has a bad reputation among patients, like it damages kidneys and eyes etc. People in this position are desperate and cling to any hope to recover from it. They look for folk healers and alternative medicine treatments and want to find something to achieve full recovery.” [Participant 5, Pharmacist, 12 years’ experience].

“Standard medications are not approved until they are tested by the Food and Drug Authority and checked for impurities. Folk healers must be supervised and have whatever they offer to people should be tested.” [Participant 14, Endocrinologist, 6 years’ experience].

### Discussing CAM use with diabetic patients

#### Frequency and nature of discussions

HCPs reported that they had never discussed or rarely discussed CAM with their diabetic patients. On the rare occasions they did, patients initiated the discussions about their CAM use as it was not part of the regular consultation. They had read or heard about the benefits of these CAM therapies for diabetes. While most participants discouraged CAM use in general, they approved of their patients using certain well-established and safe CAM therapies in rare instances. Therefore, participants acknowledged that limited discussions on CAM use with their diabetic patients would make it challenging to evaluate patients’ medication adherence or CAM-drug interactions accurately. A general practitioner participant explained that they ask the patients about anything else they use, to prevent overdosing, especially in patients with polypharmacy, i.e., patients taking multiple medications simultaneously. They described some patients talking about the types of CAM they use when they asked about other medications.

“Some patients use medications from outside pharmacy so when they come to us, we have to evaluate them. Majority of patients which we see have poly pharmacy we call it that if they use more than five medications at the same time. We ask them are you taking other things from another hospital or some other medications. Some patients replied that they use CAM.” [Participant 16, General Practitioner, 10 years’ experience].

## Barriers to effective communications around CAM

The participants provided numerous explanations in regard to why they would not broach the subject of their diabetic patients’ use of CAM during consultations. Short appointment times were cited as a barrier, as patients lack time to discuss CAM. Additionally, lack of knowledge was another commonly mentioned barrier. Participants pointed out that CAM was never part of their official medical education or professional training at any stage. On rare occasions when patients asked for consultations about CAM, healthcare providers said they had no sources or guidelines to provide professional or reliable advice. Participants explained that even if they hypothetically wanted to discuss CAM with their diabetic patients, they were restricted by the approved protocols by the Ministry of Health, which do not allow any discussion with the patients outside the scope of conventional management. Some described that HCPs would be legally liable if they advised patients on CAM use for diabetes.

“We do not have in-depth knowledge about them, and there are no scientific studies on them. The field is not motivating to conduct studies because they are not recognised anyway. If you look for information related to it, you may face legal consequences if you directed, advised, or provided medical advice to a patient on any type of complementary and alternative medicine and they experience complications or risks.” [Participant 3, Pharmacist, 6 years’ experience].

“I cannot advice. We do not have the capabilities, resources, laboratories, or sufficient experience and research. Most of the alternative medicine is promoted based on personal experience, regardless of its credibility” [Participant 7, Pharmacist, 7 years’ experience].

## Discussion

### Summary and discussion of key findings

Healthcare professional participants in this study generally had a negative attitude towards their patients’ use of CAM for managing diabetes. However, this study highlighted limited circumstances where HCPs would recommend using CAM where its safety was perceived as well-known or where they believed it would address patient concerns. Participants’ opposing views on CAM safety and effectiveness were often linked to limited evidence. Furthermore, the participants in clinical practice described challenges to ascertaining the effectiveness, referring to confounding factors such as prescribed medication adherence and patient diet and lifestyle.

Participants in this study were reluctant to dismiss the effectiveness of CAM that was associated with religious practices. However, they ignored the effectiveness of other CAM types linked to cultural (but not religious) practices, such as cauterization and folk healers, and deemed the former harmful, invasive, and ineffective and the latter ‘fraudulent’.

This study showed that discussions between HCPs and their patients about CAM were limited in the context of diabetes, which resonates with prior literature [2, 22]. A systematic review and meta-analysis of existing literature listed 84 reasons for limited discussion about CAM use between HCPs and patients in general and cited examples such as patients’ fear of the provider’s disapproval and patients perceiving disclosure of CAM use as unimportant [22]. This study provides in-depth reasons for these limited discussions and the barriers to effective communication. Many participants admitted that limited knowledge and sources of CAM information were why they did not discuss CAM with their patients. Others explained that management protocols set by health authorities [23] do not allow such discussions to occur. Participants described having limited knowledge about CAM-related side effects, and none discussed these with patients. None of the participants reported any CAM-related side effects to the National Pharmacovigilance and Drug Safety Centre, even though the Saudi Food and Drug Authority encourages reporting herbal product-related side effects [24].

This study, focusing on HCPs perspectives on CAM use among diabetic patients in Saudi Arabia, prompts a comparison with a study in Western Uganda [14]. The Uganda study explored healthcare providers’ views and patients’ reasons for seeking unconventional care, identifying barriers to diabetes management. Despite differing contexts, both studies reveal a common issue – a lack of engagement between healthcare providers and diabetic patients regarding CAM. The findings of the current study, which indicate negative perceptions due to limited knowledge and resources, parallel the concerns found in the Uganda study. Another study in Saudi Arabia focuses on herbal usage among type II diabetic patients, emphasizing a communication gap between patients and doctors about herbal self-medication [15].

#### Implications for practice

This study was conducted in Saudi Arabia, but the implications for practice based on the findings could also be applicable in other parts of the world, as diabetic patients share similar experiences with their diabetes management [2–4]. HCPs should be encouraged to discuss CAM with their diabetic patients openly, allowing further opportunities to effectively counsel patients on medication adherence and detect CAM-drug interactions or CAM-related side effects and adverse events. The study participants reported that many patients who were newly diagnosed with diabetes turned to CAM when they failed to experience immediate improvement after starting prescribed antidiabetic medication. It is crucial to have effective communication regarding medication adherence before and after blood glucose stabilization to prevent such perceptions and behaviours. Furthermore, effective communication with patients is also necessary to prevent patient exploitation and the spread of false information.

The findings of this study revealed that HCPs had limited knowledge about CAM. Participants indicated that there are limited courses on CAM directed at HCPs. Currently, CAM subjects are not widely taught in healthcare education, and available methods have been inconsistent in quantity and learning objectives [25, 26]. Incorporating CAM into healthcare education, including commonly used CAM types, would allow HCPs to form an informed attitude towards CAM use, and thus, they would be better able to counsel their patients about CAM [27]. Resources on CAM use for diabetes should be available at HCPs’ disposal when advising diabetic patients about CAM. The education and training programs should also include culturally sensitive information for HCPs and patients, as some CAM types have religious significance.

Currently, protocols set by the Ministry of Health in Saudi Arabia do not prompt discussing CAM use with diabetic patients [23]. Health authorities should consider patients’ management choices and introduce consultation protocols that are more flexible to accommodate all aspects of the disease management approaches used by patients, including CAM.

#### Implications for research

Although research on effective communication practices between patients and healthcare providers exists [28, 29], none has focused on communication-related to the use of CAM for diabetes. Further research, including those involving analysis of consultation data or through direct observations, is needed to identify the gaps and barriers to effective communications concerning CAM use [30, 31].

Due to the influence of cultural beliefs on some patients’ decisions to use CAM for diabetes management, it is necessary to conduct studies to develop and assess the provision of culturally sensitive information on CAM’s evidence-based safety and effectiveness for HCPs.

## Limitations of this study

The COVID-19 pandemic required that recruitment and interviews be conducted remotely, which limited the ability to establish rapport between participants and researchers. Face-to-face interviews would have allowed a stronger connection between participants and researchers, potentially leading to more in-depth and nuanced data collection [32]. Other studies have included practitioners outside clinical settings [33, 34], but the current study only focused on HCPs’ treating diabetic patients who visited their clinics and community pharmacies in other settings. The study was conducted in a single region of Saudi Arabia, which limits the transferability of the findings to other areas.

## Conclusion

HCPs reported limited interactions with diabetic patients concerning CAM. To optimize treatment outcomes, clinicians should consider patients’ use of CAM in areas where CAM is prevalent or holds cultural and religious significance during clinical consultations. Additionally, culturally sensitive and evidence-based information should be readily available to both diabetic patients and HCPs.

### Electronic supplementary material

Below is the link to the electronic supplementary material.

Supplementary Material 1

## Data Availability

All data in relation to this study are presented in this manuscript.
