# Supplementary figures and images for: Views of healthcare professionals on complementary and alternative medicine use by patients with diabetes: a qualitative study
Source: BMC Complement Med Ther. 2024 Feb 9;24:81. doi: 10.1186/s12906-024-04385-6 (PMC10854181; doi:10.1186/s12906-024-04385-6)

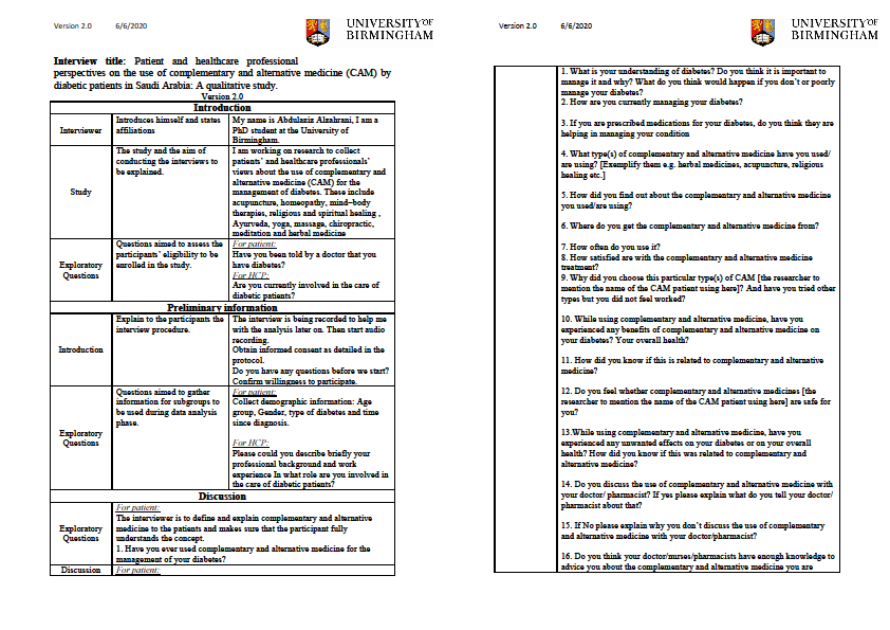

Supplement: Supplementary file 1 — Supplementary Material 1 [file 12906_2024_4385_MOESM1_ESM.png]
